# Calcium Mediated Cold Acclimation in Plants: Underlying Signaling and Molecular Mechanisms

**DOI:** 10.3389/fpls.2022.855559

**Published:** 2022-04-27

**Authors:** Zahra Iqbal, Anjuman Gul Memon, Ausaf Ahmad, Mohammed Shariq Iqbal

**Affiliations:** ^1^Molecular Crop Research Unit, Department of Biochemistry, Chulalongkorn University, Bangkok, Thailand; ^2^Department of Biochemistry, College of Medicine, Qassim University, Buraydah, Saudi Arabia; ^3^Amity Institute of Biotechnology, Amity University Lucknow, Lucknow, India

**Keywords:** calcium, calmodulin, CAMTA, cold stress, transcription factor

## Abstract

Exposure of plants to low temperatures adversely affects plant growth, development, and productivity. Plant response to cold stress is an intricate process that involves the orchestration of various physiological, signaling, biochemical, and molecular pathways. Calcium (Ca^2+^) signaling plays a crucial role in the acquisition of several stress responses, including cold. Upon perception of cold stress, Ca^2+^ channels and/or Ca^2+^ pumps are activated, which induces the Ca^2+^ signatures in plant cells. The Ca^2+^ signatures spatially and temporally act inside a plant cell and are eventually decoded by specific Ca^2+^ sensors. This series of events results in the molecular regulation of several transcription factors (TFs), leading to downstream gene expression and withdrawal of an appropriate response by the plant. In this context, calmodulin binding transcription activators (CAMTAs) constitute a group of TFs that regulate plant cold stress responses in a Ca^2+^ dependent manner. The present review provides a catalog of the recent progress made in comprehending the Ca^2+^ mediated cold acclimation in plants.

## Introduction

Plants sense and respond to distinct environmental and developmental cues *via* intricate signal transduction pathways. The signal transduction pathways comprise various protein and non-protein elements. The protein elements encompass various enzymes, receptors, and TFs, while the non-protein elements include second messengers such as Ca^2+^, cyclic AMP, cyclic GMP, inositol triphosphate, diacylglycerol, lipids, and hydrogen ions. Amongst all the reported second messenger molecules, Ca^2+^ is considered central to several signal transduction pathways (Stael et al., 2012; Sarwat et al., 2013; Kudla et al., 2018). Ca^2+^ is an essential plant macro-nutrient that is pivotal for maintaining the structural integrity of cell walls, regulating stomatal guard cells movement, growth of pollen tubes, and elongation of root hairs (Sanders et al., 2002; White and Broadley, 2003; Dodd et al., 2010). Ca^2+^ signals are elicited when a plant experiences any environmental and developmental stimuli, leading to spatial and temporal changes in Ca^2+^ ion concentration in cells. Several reviews have extensively covered different aspects of plant Ca^2+^ signaling (Costa et al., 2018; Kudla et al., 2018; Thor, 2019; Tian et al., 2020; Iqbal et al., 2021a; Pirayesh et al., 2021). Briefly, under control conditions, the levels of Ca^2+^ ions in the cell are usually low (ranging from 100 to 200 nm), but upon receiving signals to respond, the Ca^2+^ channels are transiently opened, resulting in the rapid influx of Ca^2+^ ions inside the cell. This eventually leads to an increase in cytosolic Ca^2+^ ([Ca^2+^]cyt) levels. The levels of Ca^2+^ ion inside the cell fluctuates either due to Ca^2+^ influx *via* dedicated channels or Ca^2+^ efflux *via* specific pumps (Xiong et al., 2006; Tuteja and Mahajan, 2007). In *Arabidopsis thaliana*, plasma membrane-bound Ca^2+^-permeable channels are categorized into four main families, namely, cyclic nucleotide-gated channels (CNGCs), glutamate receptor-like channels (GLRs), stretch-activated Ca^2+^ channels (OSCAs), and the MID1-complementing activity (MCA) (Romola, 2002; Kurusu et al., 2013; Jha et al., 2016; Liu X. et al., 2018). Several other Ca^2+^ channels are localized in organelles, such as endoplasmic reticulum, mitochondria, golgi body, and plant vacuole (Costa et al., 2018; Thor, 2019; He et al., 2021; Pandey and Sanyal, 2021). These include autoinhibited Ca^2+^-ATPases (ACAs), ER-type Ca^2+^-ATPases (ECAs), mitochondrial Ca^2+^ uniporter (MCU), P1-ATPases (e.g., HMA1), Ca^2+^ exchangers (CAX), two-pore channel (TPC), 1,4,5-trisphosphate receptor-like channel (InsP_3_R), 1,4,5-trisphosphate (IP_3_), cyclic ADP-ribose (cADPR)-activator ryanodine receptor-like channel (RyR), slow-activating vacuolar channel (SV), and sodium–calcium exchanger (NCX).

**FIGURE 1:**
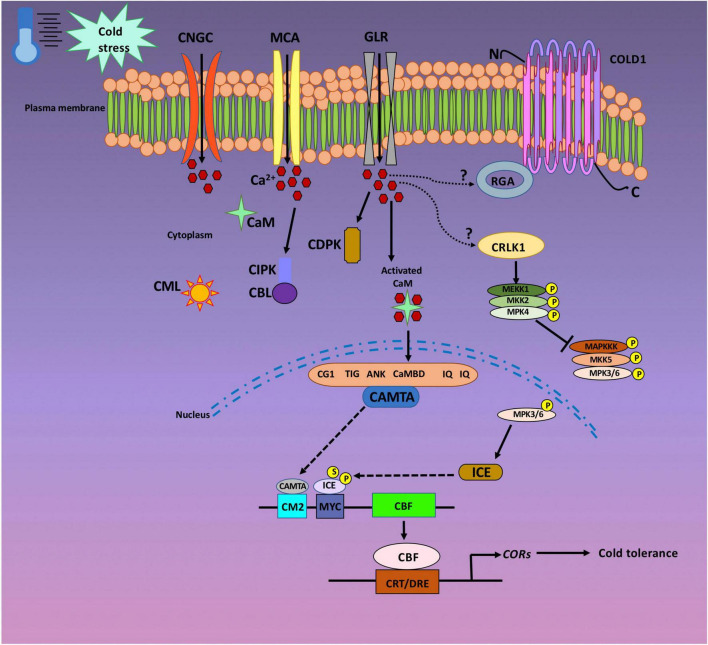
Cold stress signaling in a plant cell. The plasma membrane is considered as one of the primary target for cold sensing and eventual transmission of Ca^2+^ signals into the plant cell nuclei. Cyclic nucleotide-gated channels (CNGCs), glutamate receptor-like channels (GLRs), and the MID1-complementing activity (MCA) channels are the main plasma membrane Ca^2+^ channels that allow the entry of Ca^2+^ ions into the cytoplasm. Once the Ca^2+^ ion enters the plant cell, they are sensed by Calmodulins (CaMs) and Calmodulin-like proteins (CMLs), Calcineurin B-like proteins (CBLs), and Ca^2+^-dependent protein kinases (CDPKs). Upon cold exposure, plasma membrane associated cold sensor chilling-tolerance divergence 1 (COLD1) interacts with G protein a subunit (RGA). Ca^2+^/CaM regulated receptor-like kinase (CRLK) positively regulate cold triggered gene expression by inducing the MEKK1–MKK2–MPK4 pathway. CRLK suppress cold-induced activation of MPK3/6 and is necessary for inducer of CBF expression (ICE) accumulation. ICE proteins are stabilized by either phosphorylation (P) or sumolyation (S). Calmodulin binding transcription activators (CAMTAs) activate *C-repeat binding factor* (CBF) expression through the CM2 (CCGCGT) promoter motif. CBF proteins eventually activate the expression of various *cold-responsive (COR) genes* which confers cold tolerance in plants.

The stimuli triggered by environmental or developmental signals generates discrete Ca^2+^ signatures that are sensed and recognized by specific Ca^2+^ sensors. This cascade of events eventually results in transcriptional and metabolic responses (Perochon et al., 2011). Ca^2+^ signals are recognized by most of the Ca^2+^ sensors *via* the elongation factor hand (EF-hand) motif. Multiple EF-hand containing proteins are present in plants, and Ca^2+^ sensors represent just one of the many that translate chemical signals into an appropriate biochemical response. The EF-hand motif is represented by a conserved helix–loop–helix structure that binds to one Ca^2+^ ion. They occur in pairs as distinct domain, hence, the majority of Ca^2+^ sensors harbor two, four, or six EF-hands (Gifford et al., 2007; Perochon et al., 2011). The pairing in certain cases is generally co-operative, consequently minimizing the required Ca^2+^ signal for protein saturation. Conformational changes occur upon binding of Ca^2+^ ion to appropriate Ca^2+^ sensor. These structural changes prompt the interaction between the sensor and its target protein (TP). Three major classes of Ca^2+^ sensor families have been recognized in plants, namely, (i) Calmodulins (CaMs) and calmodulin-like proteins (CMLs), (ii) calcineurin B-like proteins (CBLs), and (iii) Ca^2+^-dependent protein kinases (CDPKs) (Hrabak et al., 2003; Batistič and Kudla, 2012). CaMs are highly conserved in eukaryotes, while CMLs, CBLs, and CDPKs had only been reported in plants and protists (Day et al., 2002; Reddy and Reddy, 2004). CaMs, CMLs, and CBLs are small protein molecules possessing a Ca^2+^ sensing domain, thereby, acting as sensor relays. They tend to bind to the downstream effector molecules in a Ca^2+^ concentration-dependent manner (Luan et al., 2002). Different from the afore-mentioned Ca^2+^ sensors, CDPKs possess an effector domain (serine/threonine protein kinase catalytic domain) along with the Ca^2+^ sensing domain. Accordingly, CDPKs act as sensor responders to directly activate and regulate their TPs upon sensing Ca^2+^ signals (Hashimoto and Kudla, 2011). Thus, the series of events: perception of stress, the opening of Ca^2+^ channels, transient changes in Ca^2+^ levels, sensing of Ca^2+^ signals by appropriate Ca^2+^ sensor, and subsequent activation of TFs for downstream molecular and biochemical outputs generates specific responses by the plant to combat the cold stress condition. One such TF is CAMTA that regulates plant responses toward cold stress in a Ca^2+^ dependent manner (Iqbal et al., 2020b). The CAMTA protein is characterized by the presence of five functional domains: CG- DNA binding motif, TAD- transcriptional activation domain, TIG- for non-specific DNA interaction, Ankyrin repeats- protein–protein interaction, CAMBD- for CaM binding. Concisely, when a plant is exposed to cold stress, the Ca^2+^ channels are opened leading to a rapid and transient influx of Ca^2+^ inside the cell. This results in an increase in ([Ca^2+^]_cyt_), which is sensed by Ca^2+^ sensor—CaM. Eventually, CaM in a Ca^2+^ dependent manner regulates the transcriptional activity of the *CAMTA* gene, withdrawing an appropriate response by the plant against cold stress. The present review summarizes the progress made in the recent years to comprehend the involvement of Ca^2+^signaling in cold stress tolerance (Figure 1).

## Calcium Sensing Network Under Cold Stress

Low temperatures lead to intricate cellular and molecular mechanisms inside plant cells *via* key components of Ca^2+^ signaling (Yuan et al., 2018b). Ca^2+^ channels play critical roles in low-temperature acclimatization of chilling-tolerant *A. thaliana* and root hair development (Hong-Bo et al., 2008). It has been proposed that Ca^2+^-permeable mechanosensitive channels MCA1 and MCA2 regulate cold-induced [Ca^2+^]cyt increase, cold tolerance, and *CBF/DREB1*-independent cold signaling. The cold-induced [Ca^2+^]cyt was lower in *mca1* and *mca2* mutants than control plants. The *mca1 mca2* double mutant compared to control were more sensitive to chilling and freezing stress (Mori et al., 2018). Additionally, vesicle membrane Ca^2+^/H^+^ antiporter, *A. thaliana calcium exchanger 1* (*AtCAX1*) is implicated in an accurate development of the cold-acclimation response by regulating the induction of *CBF/DREB1* and downstream genes (Catalá et al., 2003). Recently, Ca^2+^/cation antiporter (CaCA) superfamily proteins have been identified in *Saccharum* to play pivotal roles in environmental stresses, including cold (Su et al., 2021). Likewise, CNGC is a family of non-selective cation-conducting channels primarily localized to the plasma membrane (Zelman et al., 2012). They are implicated in thermal sensing and thermotolerance in *Arabidopsis thaliana* and mosses (Finka et al., 2012). CNGCs have been reported to play crucial roles in regulating cold tolerance in plants. *Oryza sativa OsCNGC9* transcriptional activation and phosphorylation confers enhanced chilling tolerance in rice (Wang et al., 2021). *OsCNGC9* overexpression provides increased cold tolerance, while its mutation leads to defects in cold-induced Ca^2+^ influx. Rice OsDREB1A TF is responsible for the activation of *OsCNGC9* transcription. In crux, *OsCNGC9* increases chilling tolerance by regulating cold-induced Ca^2+^ influx and [Ca^2+^]cyt elevation (Wang et al., 2021). Additionally, CNGC family has been characterized in *Chinese jujube (Ziziphus jujuba Mill.)*, and *ZjCNGC2* was reported to regulate signaling cascades in response to cold stress (Wang et al., 2020b). Further, it was shown that rice *CNGC14* and *CNGC16* are involved in promoting tolerance toward heat and chilling stresses, and are regulators of Ca^2+^ signals in response to temperature stress (Cui et al., 2020). Their homologs in *A. thaliana* (*AtCNGC2* and *AtCNGC4*) are also implicated in tolerance toward low temperature (Cui et al., 2020). CNGCs had also been implicated in modulating cold stress responses along with other biological stresses *via* Ca^2+^ signals in *Brassica oleracea* (Kakar et al., 2017), *O. sativa* (Nawaz et al., 2014), and *Nicotiana tabacum* (Nawaz et al., 2019).

The endoplasmic reticulum and plasma-membrane localized G-protein regulator CHILLING TOLERANCE DIVERGENCE1 (COLD1) coupled with RICE G-PROTEIN α SUBUNIT1 (RGA1) was reported in cold stress signaling *via* Ca^2+^ signals and electrophysiological responses in *O. sativa* (Ma Y. et al., 2015). The COLD1-RGA1 complex regulates the cold stress-driven influx of intracellular Ca^2+^, eventually resulting in the activation of *COR* (*cold regulated*) genes. It remains a subject of further evaluation whether COLD1 plays a role as a Ca^2+^-permeable channel or as a mediator promoting Ca^2+^-permeable channel activity. Taking into account another plasma membrane-bound Ca^2+^ channel—GLR—mediate Ca^2+^ fluxes across membranes and is responsive to an array of exogenous and endogenous signals in plants. *AtGLR3.4* localizes to the plasma membrane and is stimulated by cold stress in a Ca^2+^-dependent manner (Meyerhoff et al., 2005; Weiland et al., 2015). *AtGLR1.2* and *AtGLR1.3* were reported to positively regulate cold tolerance by modulating jasmonate signaling in *A. thaliana* (Zheng et al., 2018). The cold sensitivity of *glr1.2* and *glr1.3* mutants was attenuated by exogenous jasmonate treatment, while the over-expression of *GLR1.2* or *GLR1.3* led to elevated cold tolerance by enhancing endogenous jasmonate levels. Additionally, under cold stress, the expression of genes in the *CBF/DREB1* signaling pathway were lowered in *glr1.2* and *glr1.3* mutants, whereas higher in *GLR1.2* and *GLR1.3* over-expression lines (Zheng et al., 2018). Similar to the above finding, tomato *GLR3.3* and *GLR3.5* were reported to regulate cold acclimation-induced chilling tolerance by modulating apoplastic H_2_O_2_ production and redox homeostasis (Li H. et al., 2019). Next, annexins are Ca^2+^ permeable transporters that mediate the accumulation of [Ca^2+^]cyt in responses to abiotic stresses (Lee et al., 2004; Laohavisit et al., 2012; Richards et al., 2014). Recently, ANNEXIN1 was reported to regulate cold-induced Ca^2+^ influx and freezing tolerance in *A. thaliana* (Liu et al., 2021). The mutation of *AtANN1* decreased freeing tolerance, impaired cold triggered [Ca^2+^]cyt increase, and upregulated cold-responsive *CBF* and *COR* genes. The study revealed that *AtANN1* acts downstream of *OST1* in responses to cold stress (Liu et al., 2021). Furthermore, the organellar Ca^2+^ channel, *GhCAX3* gene from *Gossypium hirsutum* was characterized under various abiotic stresses, including cold. Transgenics compared to control plants were more sensitive to cold stress during seed germination. Over-expression of *GhCAX3* led to the transcript enrichment of some of the abscisic acid (ABA)-and cold-responsive genes. The study concluded that *GhCAX3* plays an imperative part in the cross-talk of cold and ABA signal transduction (Xu et al., 2013). Likewise, IP3 was reported to mediate nitric oxide (NO) triggered chilling tolerance in postharvest peach fruit (Jiao et al., 2019).

## Role of Calcium Sensors in Cold Stress

### Calmodulin and Calmodulin-Like Protein Mediated Responses Toward Cold Stress

Calmodulins and calmodulin-like protein are widely studied Ca^2+^ sensors that sense and decode rapid and transient fluctuations in the intracellular Ca^2+^ levels in response to environmental cues. In plants, CaMs and CMLs have been reported to play pivotal roles in developmental and stress biology (Zeng et al., 2015; Ranty et al., 2016; Aldon et al., 2018; Gao et al., 2019). *CaMs* and *CMLs* transcripts are induced or suppressed in response to a variety of abiotic stresses (Zeng et al., 2017; Li C. et al., 2019). Initial studies revealed that *CaM3* overexpressing lines had reduced levels of *COR* transcripts, suggestive of the fact that CaM might act as a negative regulator of cold stress (Townley and Knight, 2002). In a similar vein, *AtCaM4* had been reported to negatively regulate freezing tolerance in *A. thaliana*. The *cam4* mutants exhibited increased tolerance to freezing stress. *AtCaM4* might regulate freezing tolerance in a CBF-independent manner (Chu et al., 2018). In an interesting study, the germination of developing immature *cml39* seeds in comparison to control seeds was not sensitive to cold-stratification. Hence, it was reported that CML39 has a role in stratification-dependant seed dormancy (Midhat et al., 2018). Lately, the effect of cold stress along with other abiotic stresses was assessed for the expression of CaMs and CMLs in wild-growing grapevine *Vitis amurensis. VaCaM8* and *VaCaM10* showed significant differential expression under cold stress (4°C). Incubation at 4°C or 10°C induced the expression of six *CML* genes (*VaCML21*, *VaCML44*, *VaCML61*, *VaCML78*, *VaCML86*, and *VaCML89;* while reduced the expression of eight *CML* genes (*VaCML9a*, *VaCML48*, *VaCML57*, *VaCML75*, *VaCML82*, *VaCML85*, *VaCML92*, and *VaCML107*) (Dubrovina et al., 2019). The same group reported four alternatively spliced mRNA forms of the grapevine *CML21* gene (*CML21v1*, *CML21v2*, *CML21v3*, and *CML21v4*). All the four splice variants were highly induced under cold stress. Heterologous expression of *CML21v2* and *VaCML21v4* in *A. thaliana* increased the survival percentage of the transgenics upon freezing. Cold stress-responsive marker genes: dehydration-responsive element-binding, *AtDREB1A* and *AtDREB2A* were induced in *VaCML21v2* overexpression lines, while *AtCOR47*, *AtRD29A*, *AtRD29B*, and *AtKIN1* genes were induced in *VaCML21v4* overexpression lines after freezing stress in the transgenic *Arabidopsis* plants. Thus, it was established that *CML21* acts as a positive regulator of cold stress (Aleynova et al., 2020). Likewise, *Medicago sativa*, *MsCML46* gene encoding calmodulin-like protein confers tolerance to cold and other abiotic stress in tobacco. The *MsCML46* was upregulated in the leaves and roots after exposure to cold stress. The expression peaked after 1 h in leaves, while in roots, the expression peaked at 3 h (Du et al., 2021). In a similar vein, five *Camellia sinensis*- *CsCML* genes (*CsCML16*, *CsCML18-1*, *CsCML18-2*, *CsCML38*, and *CsCML42*) were functionally characterized under various environmental stresses. The transcript levels of *CsCML16*, *18-2*, and *42* were significantly induced by low temperature and salt stress (Ma Q. et al., 2019). Previously, *Solanum habrochaites* (cold-tolerant wild tomato) *ShCML44* gene was functionally characterized under a variety of environmental stresses, including cold stress. The *ShCML44* overexpressed plants had higher antioxidant enzymes activity, better gas exchange and water retention capacity, lower malondialdehyde (MDA) accumulation and membrane damage, reduced reactive oxygen species (ROS), and higher relative water contents (Munir et al., 2016). Very recently, *Solanum lycopersicum SlCML37* has been shown to interact with proteasome maturation factor SlUMP1 and has been reported in tomato fruit chilling stress tolerance (Tang et al., 2021). Additionally, *Medicago truncatula MtCML42* has been reported to regulate cold tolerance and flowering time (Sun et al., 2021). Further, in rice, six new putative interacting partners of OsCML16 were identified (OsLRK5a, OsDCNL2, OsWD40-139, OsGDH1, OsCIP, and OsERD2). The *in vitro* peptide-binding assays suggested that OsERD2 could bind both OsCaM1 and OsCML16, while the other five TPs specifically binded to OsCML16. Moreover, Ca^2+^ and trifluoperazine (TFP)—CaM antagonist were involved in ABA-induced transcription of *OsCML16* and its target genes. *OsCML16* and its target genes were triggered by salt, drought, and low-temperature stress (Yang et al., 2020).

Calcium/Calmodulins-regulated receptor-like kinases 1 (CRLK1) encoding a plasma membrane-associated serine/threonine kinase has been reported to play a crucial role in cold stress responses (Yang et al., 2010a,b; Furuya et al., 2013, 2014). The *crlk1* mutants compared to control plants are sensitive to freezing temperatures. The expression of cold-responsive genes, such as, *CBF1*, *RD29A*, and *COR15a* was suppressed in *crlk1* mutants, making them more susceptible to cold stress than control plants. CRLK1 protein expression is induced upon low temperature (4°C) exposures and oxidative stress (H_2_O_2_). Thus, CRLK1 is considered a positive regulator of cold stress responses in *A. thaliana*. Additionally, the Ca^2+^/CaM complex is a requisite for triggering CRLK1 kinase. It has been reported previously that an increase in CaM levels in the presence of Ca^2+^ elevates the activity of CRLK1 kinase. On the contrary, chlorpromazine (CPZ)—CaM antagonist blocked the CaM mediated CRLK1 kinase activity (Yang et al., 2010a). Explicitly, the presence of CaM-binding domain at the C-termini of CRLK1 is essential for CaM-modulated kinase activity (Yang et al., 2010a). Besides, the inducer of CBF expression 1 (ICE1) is a transcription activator and a major component of the cold response pathway as it binds with the promoters of the *C-repeat binding factor (CBF)* and *COR* genes (Tang et al., 2020). CRLK1 and CRLK2 suppress cold-induced activation of MPK3/6 and are necessary for ICE1 accumulation (Zhao et al., 2017). Hence, there exists a Ca^2+^ signaling-mediated cold-responsive pathway which is regulated by CRLK1 (Yang et al., 2010a,b).

### Calcineurin B-Like Proteins Mediated Responses Toward Cold Stress

Calcineurin B-like proteins represent a major class of Ca^2+^ binding proteins and are considered imperative relays in plant Ca^2+^ signaling pathways. CBL and CBL-interacting protein kinase (CIPK) complex are central to Ca^2+^ signaling. This complex had been reported to be implicated in a plethora of external stress signals (Kolukisaoglu et al., 2004; Yu et al., 2014; Mohanta et al., 2015). In this context, *CBL9* had been shown to negatively regulate cold tolerance *via* Ca^2+^ signaling in *A. thaliana* (Gao and Zhang, 2019). *cbl9* mutants showed enhanced freezing tolerance under cold-acclimating and non-acclimating conditions. Exposure to cold stress increased [Ca^2+^]cyt in *cbl9* mutants compared to wild type. Contrarily, ethylene glycol-bis(2-aminoethylether)-*N*,*N*,*N*′,*N*′-tetraacetic acid (EGTA)—Ca^2+^ chelator and lanthanum chloride—Ca^2+^ channel blocker significantly altered [Ca^2+^]cyt in *cbl9* mutants (Gao and Zhang, 2019). Lately, in *Camellia sinensis* (tea plant), it was shown that *CsCBL9* and *CsCIPK4/6a/6b/7/11/14b/19/20* were upregulated in both mature leaves and young shoots upon cold stress. Results of yeast two-hybrid assay demonstrated that CsCBL1 potentially interacted with CsCIPK1/10b/12 but not with CsCIPK6a/7/11/14b/20. Similarly, CsCBL9 interacted with CsCIPK1/10b/12/14b but not with CsCIPK6a/7/11/20. Thus, the study proposed distinct responses to cold stress mediated by CBL–CIPK complexes (Wang et al., 2020a). In addition, CIPKs had also been functionally characterized in *Triticum aestivum* (Deng et al., 2013), *Capsicum annuum* (Ma X. et al., 2019), *Manihot esculenta* (Mo et al., 2018), *Malus domestica* (Wang et al., 2012; Niu et al., 2018), and *Brachypodium distachyon* (Luo et al., 2018) under different environmental cues, including cold stress. *TaCIPK29* transcript increased after cold treatment (Deng et al., 2013), while *CaCIPK1* expression changed in response to cold stress (Ma X. et al., 2019). The expression of *MeCIPK7* significantly increased in roots upon cold treatment. The transcript levels of *MeCIPK10* and *13* in roots, whereas transcript levels *MeCIPK12* and *16* in leaves were also altered upon cold treatment (Mo et al., 2018). This study by Mo et al. (2018) suggested that cassava (*Manihot esculenta*) CBL–CIPK signal networks function in responses to abiotic stresses. *MdCIPK6L* ectopic expression significantly enhanced chilling tolerance in transgenic tomatoes (Wang et al., 2012), whereas the ectopic expression of *BdCIPK31* renders increased low-temperature tolerance in transgenic tobacco (Luo et al., 2018). Likewise, CBLs had been molecularly characterized under a variety of environmental stresses, including cold in *Brassica napus* (Zhang H. et al., 2014), *Brassica rapa* (Jung et al., 2017), *Stipa purpurea* (Zhou et al., 2016), and *Pyrus betulifolia Bunge* (Xu Y. et al., 2015). For *Brassica napus*, *BnaCBL1* transcripts significantly increased at 6 h of cold treatment; however, it was downregulated at 24 h. At 24 h of cold treatment, only *BnaCBL10* was slightly upregulated, and transcripts of *BnaCBL2*, *-3*, *-4* were downregulated (Zhang H. et al., 2014). For *Brassica rapa*, *BrCBL1-1* transcript levels were highly elevated (∼30-fold upregulation) after 4 h of cold treatment in one of the in-bred lines of *Brassica rapa* (Chiifu) (Jung et al., 2017). Further, overexpression of *SpCBL6* from *Stipa purpurea* increased cold tolerance and decreased drought tolerance in transgenic *A. thaliana* (Zhou et al., 2016). On similar grounds, *PbCBL1* responded to alterations in the intracellular Ca^2+^ concentrations and was induced by cold stress (Xu Y. et al., 2015).

### Calcium-Dependent Protein Kinases Mediated Responses Toward Cold Stress

Calcium-dependent protein kinases comprise a multi-gene kinase family in plants and are major regulators of developmental and stress responses in plants (Cheng et al., 2002; Valmonte et al., 2014). As already stated, CDPKs function as direct sensor responders to decode the Ca^2+^ signals (Hashimoto and Kudla, 2011). Upon sensing Ca^2+^ signals, CDPKs activate and regulate the TPs directly. Several CDPK-encoding genes are differentially expressed upon cold stress; however, their underlying molecular mechanisms remain elusive. In rice, *OsCPK17* targets the sucrose–phosphate synthase and plasma membrane intrinsic proteins and was reported in cold stress response (Almadanim et al., 2017). Additionally, *OsCPK24* inhibits glutaredoxin (*OsGrx10*), thereby, sustaining higher glutathione levels and phosphorylation. *OsCPK24* has been shown to positively regulate cold stress tolerance (Liu Y. et al., 2018). In yet another monocot plant—banana, *MaCDPK7* was shown to regulate the fruit ripening process and chilling resistance induced by heat treatment (Wang et al., 2017). Later, the *CDPK* gene family was characterized in banana for their involvement in the development, fruit ripening, and abiotic stress responses, including cold (Li et al., 2020). Genome-wide identification of the *CDPK* gene family in *Medicago truncatula* also revealed that *MtCDPK4*, *8*, *15*, *16*, and *22* transcripts were quickly elevated after 2 h of cold treatment (Zhao et al., 2021). Previously, the *CDPK* gene family had been identified and assessed for its involvement under abiotic stress conditions, including cold in *Solanum lycopersicum* (tomato; Hu et al., 2016), *Cucumis melo* (melon; Zhang et al., 2017), *Cucumis sativus* (cucumber; Xu X. et al., 2015), *zea mays* (maize; Kong et al., 2013). Moreover, in *Populus euphratica*, *PeCPK10* confers cold and drought stress tolerance. Precisely, overexpression of *PeCPK10* increased freezing tolerance in the transgenics. The expression of ABA and stress-responsive genes such as *RD29B* and *COR15A* were induced by constitutive expression of *PeCPK10* (Chen et al., 2013). In an interesting study, the roles of *VaCPK16*, *VaCPK25*, *VaCPK30*, and *VaCPK32* in secondary metabolites biosynthesis and stress resistance was studied in *V. amurensis* (grapevine) (Dubrovina et al., 2018). Overexpressing the *VaCPK30* gene conferred enhanced resistance to cold and salt stress in transgenics, whereas overexpressing *VaCPK16*, *VaCPK25*, and *VaCPK32* did not influence temperature and salt stress tolerance. Instead, the overexpression of *VaCPK16* and *VaCPK32* enhanced stilbene accumulation in *V. amurensis* cell cultures (Dubrovina et al., 2018). Earlier the same group had reported the involvement of *VaCPK20* in cold and drought stress response pathways (Dubrovina et al., 2015). On similar lines in *Zea mays*, *ZmCPK1* was reported as a negative regulator of cold stress signaling in maize (Weckwerth et al., 2015). *ZmCPK1* displayed Ca^2+^-independent protein kinase activity. The expression of *ZmCPK1* increased, while the expression of *ZmCPK25* decreased upon cold stress (Weckwerth et al., 2015). Recently, *Malus domestica* (apple) *MdCPK1a* gene was reported to enhance tobacco cold resistance *via* scavenging ROS accumulation (Dong et al., 2020). The underlying mechanism of cold resistance through the involvement of *MdCPK1a* was further investigated. The *MdCPK1a* tobacco transgenics had a better survival ratio and root length when subjected to cold stress. The superoxide dismutase (SOD), peroxidase (POD), and catalase (CAT) activities were higher, while electrolyte leakages (EL), MDA content, and ROS were lower. This was suggestive of the fact that the transgenics underwent less chilling injury than control plants (Dong et al., 2020). Thus, Ca^2+^ signaling plays a pivotal part in cold acclimation in plants (Table 1).

**TABLE 1:** Calcium signaling components in cold stress acclimation in plants.

Gene	Ca^2+^ component	Species	References
*MCA1 and MCA2*	Ca^2+^ channel	*Arabidopsis thaliana*	Mori et al., 2018
*AtCAX1*	Ca^2+^ channel	*Arabidopsis thaliana*	Catalá et al., 2003
*Ca^2+^/cation antiporter*	Ca^2+^ channel	*Saccharum*	Su et al., 2021
*CNGC9*	Ca^2+^ channel	*Oryza sativa*	Wang et al., 2021
*ZjCNGC2*	Ca^2+^ channel	*Ziziphus jujuba Mill*	Wang et al., 2020b
*CNGC14 and CNGC16*	Ca^2+^ channel	*Oryza sativa*	Cui et al., 2020
*AtCNGC2 and AtCNGC4*	Ca^2+^ channel	*Arabidopsis thaliana*	Cui et al., 2020
*AtGLR3.4*	Ca^2+^ channel	*Arabidopsis thaliana*	Meyerhoff et al., 2005; Weiland et al., 2015
*AtGLR1.2 and AtGLR1.3*	Ca^2+^ channel	*Arabidopsis thaliana*	Zheng et al., 2018
*GLR3.3 and GLR3.5*	Ca^2+^ channel	*Solanum*	Li H. et al., 2019
*ANNEXIN1*	Ca^2+^ channel	*Arabidopsis thaliana*	Liu et al., 2021
*GhCAX3*	Ca^2+^ channel	*Gossypium hirsutum*	Xu et al., 2013
*CaM3*	Ca^2+^ sensor	*Arabidopsis thaliana*	Townley and Knight, 2002
*CaM4*	Ca^2+^ sensor	*Arabidopsis thaliana*	Chu et al., 2018
*CML39*	Ca^2+^ sensor	*Arabidopsis thaliana*	Midhat et al., 2018
*VaCaM8 and VaCaM10*	Ca^2+^ sensor	*Vitis amurensis*	Dubrovina et al., 2019
*VaCML21*, *VaCML44*, *VaCML61*, *VaCML78*, *VaCML86*, *and VaCML89*	Ca^2+^ sensor	*Vitis amurensis*	Dubrovina et al., 2019
*CML21v1*, *CML21v2*, *CML21v3*, *and CML21v4*	Ca^2+^ sensor	*Vitis amurensis*	Aleynova et al., 2020
*MsCML46*	Ca^2+^ sensor	*Medicago sativa*	Du et al., 2021
*CsCML16*, *18-2*, *and 42*	Ca^2+^ sensor	*Camellia sinensis*	Ma Q. et al., 2019
*ShCML44*	Ca^2+^ sensor	*Solanum habrochaites*	Munir et al., 2016
*SlCML37*	Ca^2+^ sensor	*Solanum lycopersicum*	Tang et al., 2021
*MtCML42*	Ca^2+^ sensor	*Medicago truncatula*	Sun et al., 2021
*CRLK1*	Ca^2+^ sensor	*Arabidopsis thaliana*	Yang et al., 2010a,b
*CBL9*	Ca^2+^ sensor	*Arabidopsis thaliana*	Gao and Zhang, 2019
*CsCBL9 and CsCIPK4/6a/6b/7/11/14b/19/20*	Ca^2+^ sensor	*Camellia sinensis*	Wang et al., 2020a
*TaCIPK29*	Ca^2+^ sensor	*Triticum aestivum*	Deng et al., 2013
*CaCIPK1*	Ca^2+^ sensor	*Capsicum annuum*	Ma X. et al., 2019
*MeCIPK7*	Ca^2+^ sensor	*Manihot esculenta*	Mo et al., 2018
*MdCIPK6L*	Ca^2+^ sensor	*Malus domestica*	Wang et al., 2012
*BdCIPK31*	Ca^2+^ sensor	*Brachypodium distachyon*	Luo et al., 2018
*BnaCBL*	Ca^2+^ sensor	*Brassica napus*	Zhang H. et al., 2014
*BrCBL1-1*	Ca^2+^ sensor	*Brassica rapa*	Jung et al., 2017
*SpCBL6*	Ca^2+^ sensor	*Stipa purpurea*	Zhou et al., 2016
*PbCBL1*	Ca^2+^ sensor	*Pyrus betulifolia Bunge*	Xu Y. et al., 2015
*OsCPK17*	Ca^2+^ sensor	*Oryza sativa*	Almadanim et al., 2017
*OsCPK24*	Ca^2+^ sensor	*Oryza sativa*	Liu Y. et al., 2018
*MaCDPK7*	Ca^2+^ sensor	*Musa acuminata* *cv.Cavendish*	Wang et al., 2017; Li et al., 2020
*MtCDPK4*, *8*, *15*, *16*, *and 22*	Ca^2+^ sensor	*Medicago truncatula*	Zhao et al., 2021
*PeCPK10*	Ca^2+^ sensor	*Populus euphratica*	Chen et al., 2013
*VaCPK30*	Ca^2+^ sensor	*Vitis amurensis*	Dubrovina et al., 2018
*VaCPK20*	Ca^2+^ sensor	*Vitis amurensis*	Dubrovina et al., 2015
*ZmCPK1*	Ca^2+^ sensor	*Zea mays*	Weckwerth et al., 2015
*MdCPK1a*	Ca^2+^ sensor	*Malus domestica*	Dong et al., 2020
*CAMTA3*	TF	*Arabidopsis thaliana*	Doherty et al., 2009; Kim et al., 2013, Kidokoro et al., 2017; Kim et al., 2017
*CAMTA5*	TF	*Arabidopsis thaliana*	Kidokoro et al., 2017

### AtSR/CAMTA Regulated Transcription Under Cold Stress

Upon perception of cold stress, Ca^2+^ signals are elevated, which might direct Ca^2+^ to either repress or activate the activity of Ca^2+^ responding protein. Similarly, the interaction of Ca^2+^ with Ca^2+^ sensors either suppresses or enhances the binding to a TF. Depending upon whether the TF itself is a repressor or activator, the transcription of the target gene is repressed or activated. Ca^2+^/CaM dependent TFs relay cold-induced Ca^2+^ transients to transcriptional reprograming. CAMTAs are one such group of TFs that regulate plant cold stress responses in a Ca^2+^-dependent manner. CAMTA proteins have been stipulated to play a direct link between Ca^2+^ signals and cold acclimation (Eckardt, 2009). CAMTAs also known as signal responsive (SR) protein (Yang and Poovaiah, 2000) or EICBP (ethylene-induced CaM-binding proteins) (Reddy et al., 2000) is a well-characterized CaM dependent TF that regulates gene expression by binding to the signature “CGCG” DNA motif (Galon et al., 2008; Du et al., 2009; Yuan et al., 2018a). Furthermore, CBF cold response pathway plays a pivotal role in cold acclimation (Shi et al., 2018). It is characterized by rapid cold induction of genes encoding the *CBF1-3* TFs, followed by the expression of the *CBF* gene regulon. The CRT/DRE *cis*-element is recognized by the CBF protein and is characterized by the presence of a conserved CCGAC sequence. The CCGAC sequence is present in the 1000 bp upstream region of a subset of *COR* genes (Stockinger et al., 1997; Gilmour et al., 1998; Shi et al., 2018; Liu et al., 2019). The *cis* and *trans*-acting factors implicated in the expression of *CBF2* were studied by Doherty et al. (2009). Seven conserved DNA motifs (CM1 to 7) were identified in the promoters of *CBF2* and *ZAT12* (cold-induced genes). CM4 and CM6 have negative regulatory activity, while CM2 has both negative and positive activity. The study also revealed that CAMTA3 binds to the CM2 motif and is a positive regulator of *CBF2* expression. Moreover, *camta1 camta3* double mutant plants were impaired in freezing tolerance. This study exhibited a novel role of CAMTA in cold acclimation and provided a plausible link of low-temperature Ca^2+^ and CaM signaling with cold-regulated gene expression (Doherty et al., 2009). Later, CAMTA3 and CAMTA5 were reported to respond to a rapid decrease in temperature and induce the expression of *DREB1s* (Kidokoro et al., 2017). Additionally, contrary to *circadian clock associated1* and *late elongated hypocotyl* genes that modulate *DREB1* expression only during the day, CAMTA3 and CAMTA5 function both during the day and night (Kidokoro et al., 2017).

Salicylic acid (SA) has a central role in transcriptional machinery at low temperatures (Scott et al., 2004). However, accumulated SA did not influence cold tolerance in *atsr1* (also referred as CAMTA3) (Kim et al., 2013). CAMTA1 and CAMTA2 in combination with CAMTA3 induced transcripts of *CBF1*, *CBF2*, and *CBF3* at 2 h and enhanced plant freezing tolerance. Additionally, CAMTA1, CAMTA2, and CAMTA3 work simultaneously to inhibit SA biosynthesis at warm temperatures (22°C). However, the SA levels increased in plants exposed to low-temperatures for more than one week. The study revealed that the isochorismate synthase (ICS) pathway is involved in chilling-induced SA biosynthesis. The accumulation of *ICS1*, *CBP60g*, and *SARD1* transcripts were suppressed at warm temperatures by these three CAMTAs, but not at low temperatures (Kim et al., 2013). The analysis of upstream regions to the transcription start site (TSS) in wound-induced genes indicated the presence of rapid stress response DNA element (RSRE), CGCGTT. Moreover, promoter activity assay depicted that luciferase activity level induced by cold stress was lower in *camta3* mutants than control plants (Benn et al., 2014). The study revealed that CAMTA3 modulates cold tolerance in *A. thaliana via* the regulation of genes that harbor RSRE elements in their promoters (Benn et al., 2014). Another interesting study found that heptahelical protein 2 (HHP2) interacts with CBF upstream regulators, such as ICE1, ICE2, and CAMTA3 (Lee and Seo, 2015). At low-temperatures, MYB96 (R2R3-type MYB TF) induced the *HHP* genes (Lee and Seo, 2015). This suggests that a cross-wired mesh of pathways exist that incorporates Ca^2+^ signaling to regulate cold stress tolerance through CAMTA3. Kim et al. (2017) revealed that the IQ motifs in *AtCAMTA3* (residues 850–875) are necessary for its activity (Kim et al., 2017). Post-translational modifications (phosphorylation or dephosphorylation) play imperative part in *AtCAMTA3* mediated response to environmental cues. S454 and S964 were identified as two putative phosphorylation sites in AtCAMTA3 protein (Jones et al., 2009). The *camta1 camta3* double mutants complemented with mutated AtCAMTA3 protein, S454A and S964A (phosphorylation sites of AtCAMTA3) were partially restored to control plants. Moreso, the suppression of SA biosynthesis in the mutants was compromised, suggestive of the fact that phosphorylation is necessary for the full functionality of *AtCAMTA3* (Kim et al., 2017). It is well reported that CAMTA3 is a defense repressor. CAMTA3 is degraded to trigger SA-mediated immune response during pathogen incursion (Galon et al., 2008; Poovaiah et al., 2013; Zhang L. et al., 2014; Fromm and Finkler, 2015; Kim et al., 2017). Intriguingly, SA-mediated signaling pathways also cross-talk with pathways implicated in long-term cold treatments (4°C, 2 weeks) (Kurepin et al., 2013; Miura and Tada, 2014). Nonetheless, AtCAMTA3 protein is also accumulated at low temperatures (Kim et al., 2017). These observations suggest that a complex mesh of networks intersect with each other to overcome the *AtCAMTA*3 suppression of the SA signaling pathway. Very recently, evolution analyses of CAMTA genes in 112 plant species were performed to study its enhancing effect on cold tolerance (Xiao et al., 2021). Thus, CAMTAs *via* Ca^2+^/CaM signaling has an intersecting role in imparting cold tolerance to plants.

## Conclusion and Future Perspective

The underpinning mechanisms of cold signaling pathways and genes implicated in cold stress have been extensively studied in the past few years. Different signaling pathways converge to allow plants cope with cold stress. Perception of cold stress by the plant is contemplated to be the first event for the induction of Ca^2+^ transients (Ma Y. et al., 2015). The cold stress-triggered Ca^2+^ transients are generated *via* a number of Ca^2+^ channels and/or Ca^2+^ pumps. These Ca^2+^ transients are relayed and decoded by a variety of Ca^2+^ sensors to regulate gene expression and subsequently confer cold tolerance to plants (Ma Y. et al., 2015; Mori et al., 2018). Considerable advancements have been made to comprehend the underlying components of the Ca^2+^ signaling network, such as, Ca^2+^–CBL–CIPK, CDPK, and Ca^2+^–CaM–CAMTA (Weckwerth et al., 2015; Kidokoro et al., 2017; Wang et al., 2020a). Moreover, plant cold tolerance is an intricate process involving dissecting signal transduction pathways. It remains elusive how other signaling pathways intersect with Ca^2+^ signaling pathways to confer cold tolerance in plants. It is still a challenge to deeply decipher the role of Ca^2+^ signals in the cold stress tolerance mechanism and to ascertain whether cold stress-triggered Ca^2+^ transients exist in the cell nucleus. Cutting edge techniques such as multi-omics (Iqbal et al., 2021b), CRISPR/cas9 gene-editing systems (Iqbal et al., 2020a), and sensitive Ca^2+^ imaging (Grenzi et al., 2021) can prove to be potent tools to determine the un-discovered aspects of Ca^2+^ signaling pathways. Thus, future research should focus on deciphering the key converging and diverging pathways pivotal to Ca^2+^ mediated cold signaling. Further, gaining in-depth insights as to how Ca^2+^ signatures are induced and decoded in response to cold stress can help better comprehend the involvement of Ca^2+^ ion in cold stress signaling. Nonetheless, efforts should be made to identify low-temperature sensors using biological methods in combination with biochemical and biophysical approaches.

## Author Contributions

ZI drafted and wrote the manuscript. AGM and AA critically revised the manuscript for consistency and content. MSI conceptualized the idea and reviewed the manuscript. All authors reviewed and approved the final version of the manuscript.

## Conflict of Interest

The authors declare that the research was conducted in the absence of any commercial or financial relationships that could be construed as a potential conflict of interest.

## Publisher’s Note

All claims expressed in this article are solely those of the authors and do not necessarily represent those of their affiliated organizations, or those of the publisher, the editors and the reviewers. Any product that may be evaluated in this article, or claim that may be made by its manufacturer, is not guaranteed or endorsed by the publisher.
